# Random lasing and replica symmetry breaking in GeO_2_-PbO-MgO glass–ceramics doped with neodymium

**DOI:** 10.1038/s41598-022-23893-4

**Published:** 2022-11-14

**Authors:** Josivanir G. Câmara, Davinson M. da Silva, Luciana R. P. Kassab, Manoel L. Silva-Neto, Guillermo Palacios, Cid B. de Araújo

**Affiliations:** 1 Departamento de Engenharia Elétrica, Escola Politécnica, Universidade de São Paulo, São Paulo, SP 05508-970 Brazil; 2 Faculdade de Tecnologia de São Paulo, Pça Cel. Fernando Prestes, 30, São Paulo, SP 01124-060 Brazil; 3 Departamento de Física, Universidade Federal de Pernambuco, Recife, PE 50670-901 Brazil; 4 Present Address: Department of Chemistry, University of Toronto, Toronto, ON M5S 3H6 Canada

**Keywords:** Materials for optics, Lasers, LEDs and light sources, Optics and photonics, Lasers, LEDs and light sources

## Abstract

We investigated the random lasing process and Replica Symmetry Breaking (RSB) phenomenon in neodymium ions (Nd^3+^) doped lead-germanate glass–ceramics (GCs) containing MgO. Glass samples were fabricated by conventional melt-quenching technique and the GCs were obtained by carefully devitrifying the parent glasses at 830 °C for different time intervals. The partial crystallization of the parent glasses was verified by X-ray diffraction. Photoluminescence (PL) enhancement of $\approx$ 500% relative to the parent glasses was observed for samples with a higher crystallinity degree (annealed during 5 h). Powders with grains having average size of 2 µm were prepared by griding the GCs samples. The Random Laser (RL) was excited at 808 nm, in resonance with the Nd^3+^ transition ^4^I_9/2_ → {^4^F_5/2_, ^2^H_9/2_}, and emitted at 1068 nm (transition ^4^F_3/2_ → ^4^I_11/2_). The RL performance was clearly enhanced for the sample with the highest crystallinity degree whose energy fluence excitation threshold (EFE_th_) was 0.25 mJ/mm^2^. The enhanced performance is attributed to the residence-time growth of photons inside the sample and the higher quantum efficiency of Nd^3+^ incorporated within the microcrystals, where radiative losses are reduced. Moreover, the phenomenon of Replica Symmetry Breaking (RSB), characteristic of a photonic-phase-transition, was detected by measuring the intensity fluctuations of the RL emission. The Parisi overlap parameter was determined for all samples, for excitation below and above the EFE_th_. This is the first time, for the best of the authors knowledge, that RL emission and RSB are reported for a glass–ceramic system.

## Introduction

Laser action in disordered media, without optical cavities, has been the object of intense theoretical and experimental studies since the pioneering work of Letokhov^1–11^. In this kind of laser systems, currently referred as Random Lasers (RLs), the feedback mechanism contributing for optical amplification is not achieved by a well-engineered optical cavity as in conventional lasers. Instead, optical feedback is accomplished by light scattering due to refractive index inhomogeneities within a disordered medium^12,13^.

RLs were reported for several systems up to now. For example, the disordered scattering particles may be embedded within the gain medium, such as in laser-dyes liquid solutions containing high refractive index particles in suspensions^14,15^. The dyes may also be incorporated into solid matrices, such as polymer membranes^16^, biological tissues^17^, and glasses produced by sol–gel^18^, among others.

Random lasing was also extensively reported for rare-earth ions (REI) doped crystalline powders^19–25^. In these systems the particles act both as gain medium and as scatterers. In particular, random lasing can also be obtained from REI doped optical fibers, where the feedback may be obtained due to light reflections in random Bragg gratings written in fibers with nonuniform refractive indices^26,27^, or fibers with phase separated glass cores^28^.

Curiously, RL reports based on glassy particles doped with REI are very scarce. Several years ago, an upconversion RL emitting in the UV, was reported based on a fluoroindate glass-powder doped with neodymium ions^29^. More recently, we demonstrated RL action in neodymium (Nd^3+^) doped zinc-tellurite glass powders^30^. The RL feedback mechanism was provided by the light reflections on the glassy grains-air interfaces.

Works on RL in glass–ceramics (GCs) are rare as well^31,32^. Nevertheless, GCs are interesting media for photonic devices, since they can withstand high power excitations and have a high thermal threshold. Furthermore, GCs can be heavily doped with rare-earth ions to alter its emission characteristics^31^. Despite that, to the best of our knowledge, the present article is the first report of a RL based on Nd^3+^ doped glass–ceramics. Our aim was to evaluate and characterize the influence of the crystallization degree of the GCs powder on the performance of RLs. For this research, the choice of the parent lead-germanate glass was made for several reasons discussed below.

Lead-germanate glasses are strong candidates for RLs operation because they present high solubility for REI, high refractive index (~ 2.0), high transmittance window (400–5000 nm)^33^, and a high resistance to optical damage^34^. Addition of MgO in the glass composition contributed as an intermediate oxide in the glass structure, acting not only as a modifier, but also as a network former^35,36^.

Besides the basic characterization of the RLs, the statistical study of the intensity fluctuations was conducted in this work. This was motivated by several works^37–43^ demonstrating that the RL transition is a *photonic phase-transition*, analogous to the paramagnetic to spin-glass phase-transition in disordered magnetic materials^44^. In RLs this phase-transition is characterized by a Replica Symmetry Breaking (RSB) where intensity fluctuations are highly correlated. However, although RSB has been identified and studied in RLs based on liquids, crystalline powders and optical fibers, the observation of RSB in GCs was not reported.

## Results and discussion

### Morphological, structural, and thermal analysis

**Figure 1:**
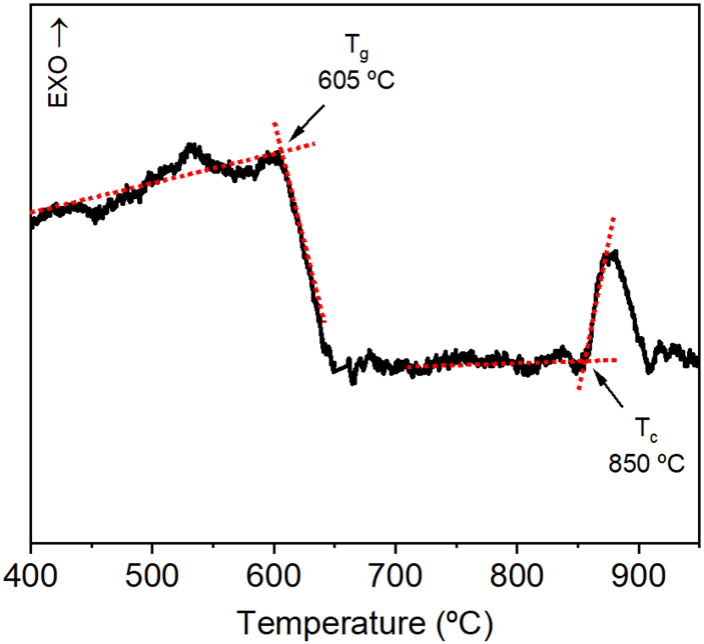
DSC of GPM (10 wt% of Nd_2_O_3_) glass sample at heating rate of 10 °C/min (T_g_ = glass transition temperature, T_c_ = crystallization temperature).

Differential Scanning Calorimetry (DSC) results for the Nd^3+^ doped GPM powder, obtained before the annealing process to prepare GCs, are shown in Fig. 1. A typical glass-transition is observed at 605 °C. Additionally, the thermogram shows an exothermic peak with an onset temperature of about 850 °C. This feature is attributed to the growth of crystalline phases within the glass. A temperature, slightly under the onset of crystallization, at 830 °C, was used in this work to induce a controlled crystallization of the samples, with the aim to obtain GCs with different degrees of crystallization. A temperature slightly under 850 °C was used to minimize the adherence of the samples to the crucible used for the thermal annealing.

**Figure 2:**
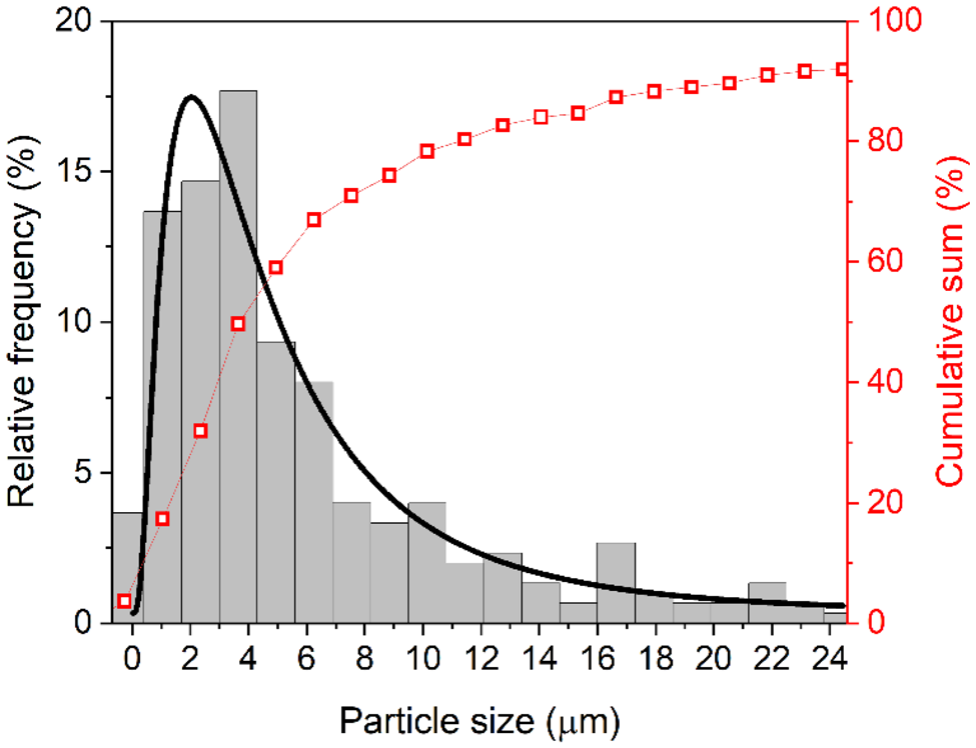
Particle size distribution for the sample GPM5 (glass–ceramic powder annealed for 5 h at 830 °C).

Figure 2 exhibits the particle size distribution of Nd^3+^ doped GPM glass–ceramic powder annealed for 5 h at 830 °C (GPM5) obtained from optical microscopy measurements. The particles average size was $\approx$ 2 µm, but it was observed a small fraction of larger particles, with dimensions up to $\approx$ 24 µm. The other samples present similar sizes distribution because the grains were collected after sedimentation of coarser particles mixed with isopropyl alcohol as described in “Optical characterization”.

**Figure 3:**
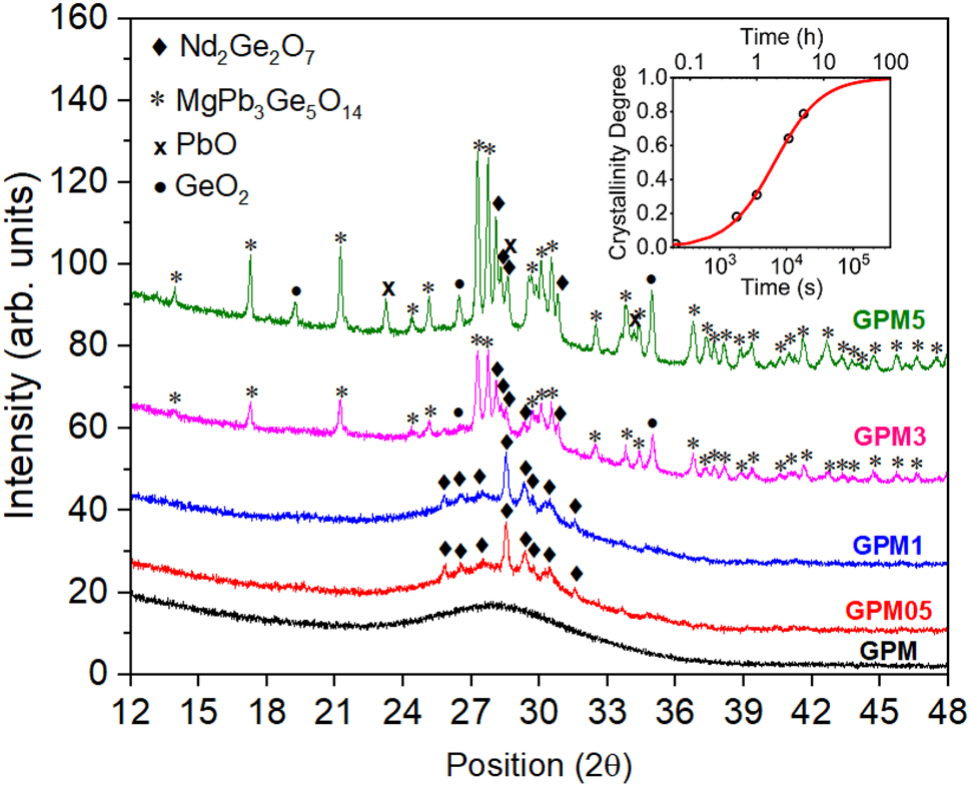
X-ray diffraction for Nd^3+^ doped samples GPMx (x = 0.5, 1, 3, 5). The inset presents the crystallinity degree as a function of annealing time.

Figure 3 shows X-ray diffractograms obtained for Nd^3+^ doped glass and GC samples. Notice that the GPM sample, not submitted to annealing, did not present evidence of crystallization. However, samples annealed at 830 °C for 0.5 h and 1 h show sharp crystallization peaks attributed to the growth of nanocrystalline Nd_2_Ge_2_O_7_ phase (PDF: 42-0208). On the other hand, GPM3 powder presents additional peaks related to the presence of MgPb_3_Ge_5_O_14_ (PDF: 39-1265) and GeO_2_ (PDF: 36-1463). Finally, in the GPM5 powder, peaks related to the growth of PbO (PDF: 3-0600) crystalline phase were also present. Scherrer equation indicated that the crystallite sizes grown in the sample had dimensions of about 50 nm.

The kinetics of the isothermal crystallization of the lead-germanate glass may be described using the Johnson–Mehl–Avrami-Komogorov (JMAK) equations^45^. In this context, the volume fraction, $x\left(t\right),$ of a crystal grown from the glass-phase during isothermal reactions can be described by the expression:

1 $$x\left(t\right)=1-\mathrm{exp}(-K{t}^{n})$$

where *n* is the Avrami exponent and *K* is the crystallization rate constant, expressed as:

2 $$K\left(T\right)={K}_{0}\cdot \mathrm{exp}\left(-\frac{{E}_{eff}}{{k}_{b}T}\right)$$

where ${E}_{eff}$ is the effective activation energy describing the overall crystallization process, ${K}_{0}$ is the isothermal JMAK parameters, *T* is the heating temperature, and ${k}_{b}$ is the Boltzmann constant. Both *n* and *K* reflect the nucleation and growth mechanisms of the sample.

The plot of the crystallinity degree as a function of time (inset of Fig. 3) presented a typical sigmoidal curve, as expected from the JMAK model. The general equation for the Avrami exponent is $n=a+bc$, where *a* is the nucleation index ($a=0$ for zero nucleation rate during phase change transformation, $0<a<1$ for a decreasing nucleation rate, a = 1 for a constant nucleation rate, and $a>1$ for an increasing nucleation rate). The parameter $b$ is the dimensionality of the crystal grown (*b* = 1 for 1D, *b* = 2 for 2D and *b* = 3 for 3D crystal) and *c* is the growth index (*c* = 0.5 for diffusion-controlled growth and *c* = 1 for interface-controlled growth)^42^. In the present case, the Avrami exponent was obtained from the fitting of the experimental data to the JMAK model (Eq. 2) and $n$ was found to be approximately 0.89. In this case, the values for *b* and *c* must be 1 and 0.5, respectively, which means that the dimensionality of the crystals were 1D and the growth process was diffusion-controlled. The parameter $a$ is 0.39 which corresponds to a decreasing nucleation rate as a function of time^46^. The effective activation energy, ${E}_{eff},$ was not estimated in this work since additional crystallinity degree versus annealing time curves for other temperatures would be necessary. Nevertheless, the investigation of the details concerning the crystals growth kinetics were out of the scope of the present work.

### Optical characterization

**Figure 4:**
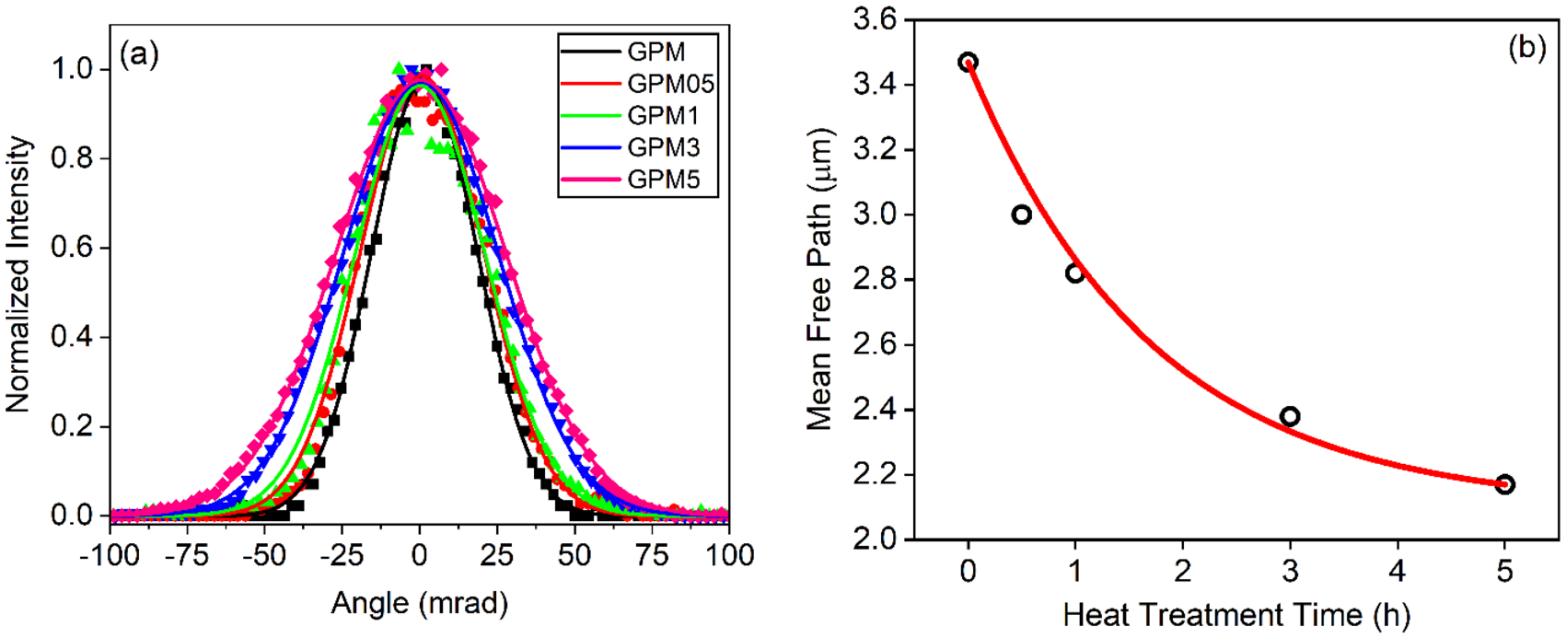
(**a**) CBS cones of scattered light intensity (at 1064 nm) as a function of detection angles for all samples prepared. (**b**) Mean-free-path as a function of annealing time.

Coherent Back Scattering (CBS) results are shown in Fig. 4a. The transport mean-free-path $({l}_{t})$ as a function of the annealing times were determined using ${l}_{t}=0.7\frac{\lambda }{2\pi W}$ as described in “Optical characterization” and are shown in Fig. 4b. We observe an expressive reduction of the mean-free-path, as the annealing time increases. Hence, it is evident that crystallites that are grown within the host glass are also acting as scattering media. This is an interesting result from a materials characterization point of view, since our results show that CBS measurements may also be a suitable technique to access the crystallization behavior of transparent solids, such as glasses or polymers.

**Figure 5:**
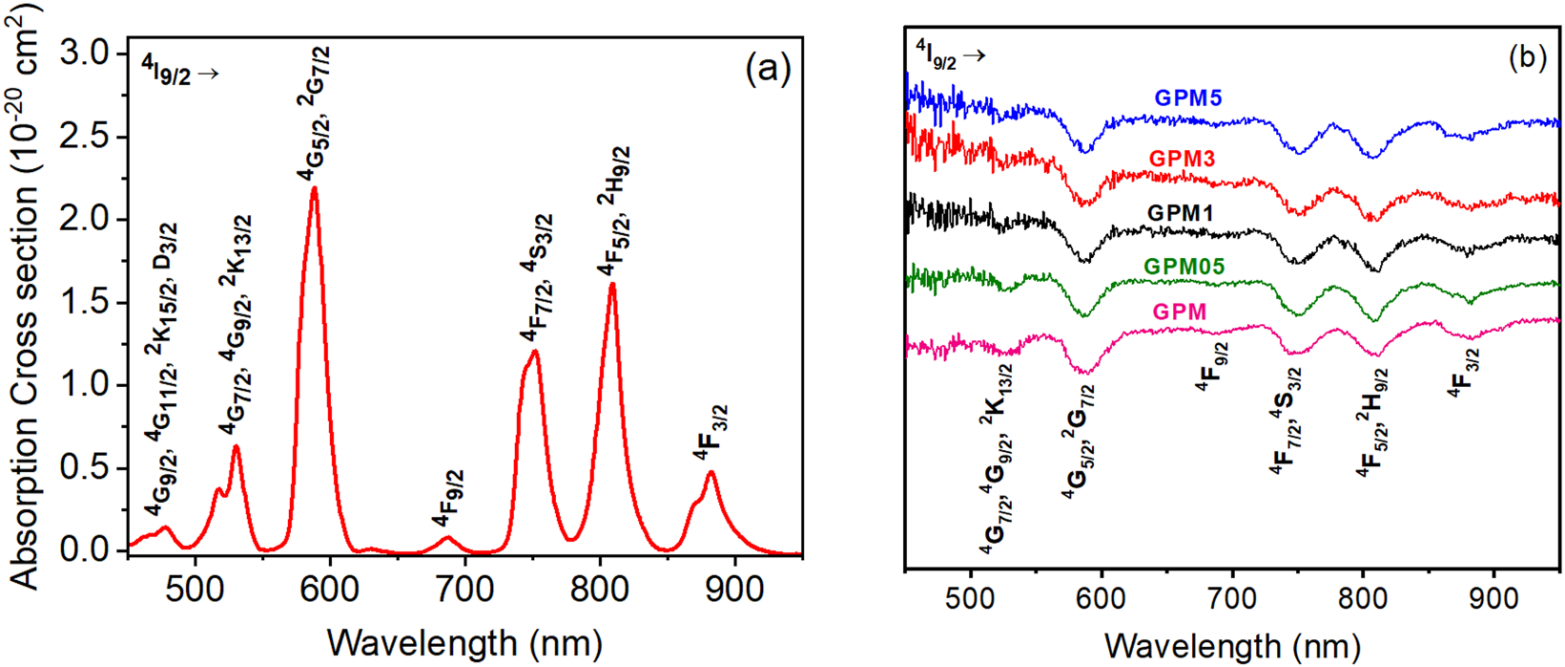
(**a**) Absorption cross section of the precursor GPM glass with neodymium ions. The absorption bands are due to transitions from the ground state to the Nd^3+^ excited states. (**b**) Diffuse reflectance spectra of all prepared powders. The spectra are shifted in the vertical axis to prevent overlap.

Figure 5a shows the absorption cross section of the precursor GPM glass doped with 10 wt% Nd_2_O_3_, and Fig. 5b shows the diffuse reflectance spectrum of the glass and GCs powders. Absorption peaks could be observed at about 532 nm, 585 nm, 690 nm, 750 nm, 808 nm, and 880 nm, which are related to the Nd^3+^ transitions ^4^I_9/2_ → {^4^G_7/2_, ^2^G_9/2_, ^2^K_13/2_}, ^4^I_9/2_ → {^4^G_5/2_, ^2^G_7/2_}, ^4^I_9/2_ → ^4^F_9/2_, ^4^I_9/2_ → {^4^F_7/2_, ^4^S_3/2_}, ^4^I_9/2_ → {^4^F_5/2_, ^2^H_9/2_}, and ^4^I_9/2_ → ^4^F_3/2_, respectively. Evidencing the incorporation of neodymium in the trivalent form (Nd^3+^) in the glass and glass–ceramics studied.

**Figure 6:**
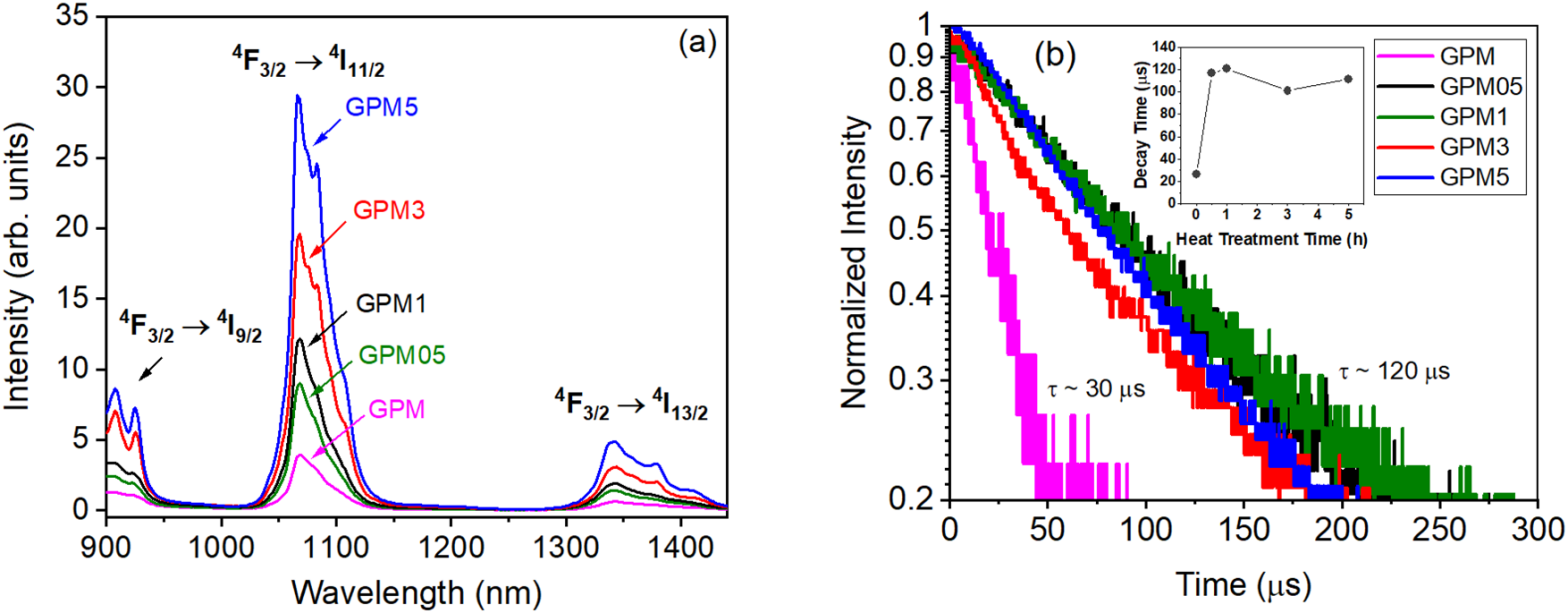
(**a**) PL spectra of the powders showing the Nd^3+^ transitions ^4^F_3/2_ → ^4^I_9/2_, ^4^F_3/2_ → ^4^I_11/2_ and ^4^F_3/2_ → ^4^I_13/2_. (**b**) Temporal behavior of the 1068 nm PL signal, that corresponds to transition (^4^F_3/2_ → ^4^I_11/2_), for all samples.

The PL spectra and the temporal behavior of the samples excited with low pump intensity using a CW laser at 808 nm are shown in Fig. 6. Notice in Fig. 6a that the emission band at ~ 1068 nm (due to transitions ^4^I_9/2_ → {^4^F_5/2_, ^2^H_9/2_}) grows as the annealing time increases. The sample annealed for 5 h showed a PL intensity ~ 500% higher in comparison with the sample that was not subjected to the crystallization process. The temporal behavior of the PL emission at 1068 nm of the glass and GCs is shown in Fig. 6b. The decay lifetime rises from 30 µs for the GPM glass to approximately 120 µs for the GCs samples (see inset in Fig. 6b).

**Figure 7:**
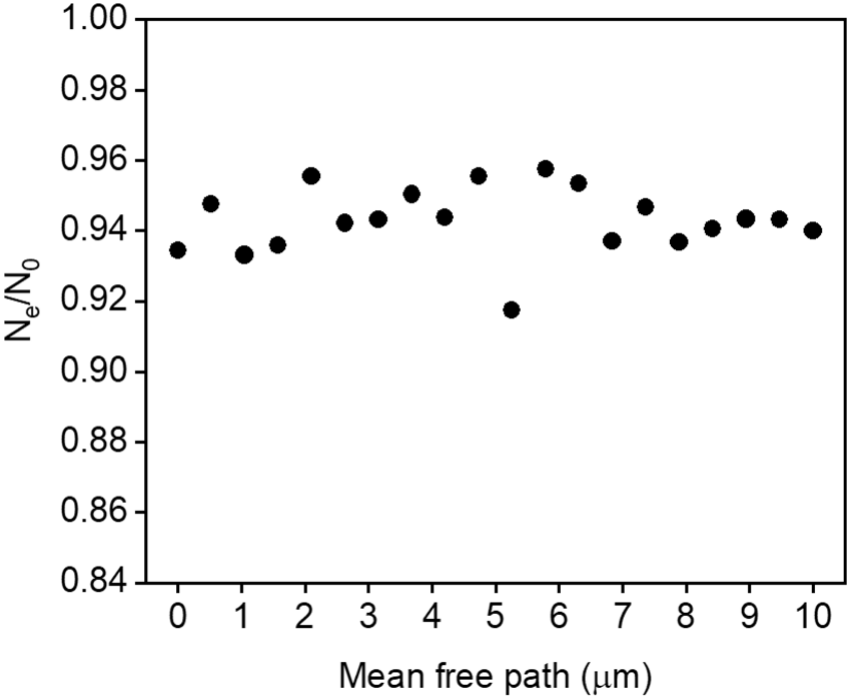
Monte Carlo Simulation of the ratio between spontaneous photons that can leave the sample to spontaneous photons that are generated within the host material as a function of the mean-free-path.

The PL enhancement observed in Fig. 6a is correlated to the degree of crystallization of the samples. To verify if the photons mean-free-path was correlated to the PL results, a Monte Carlo simulation was performed. The algorithm used considered that each incident pump photon would follow a 3D random walk inside the sample, with an exponential distribution for the path lengths between successive scattering events, with the mean-free-path within the range of transport lengths determined from the CBS measurements. There were no constraints for the size of the simulation box, except for the plane of incidence of photons, located at z = 0. To simulate an incident photon on the sample, the initial polar and azimuthal angles of the displacement vector of the photon are chosen to be (θ_0_, φ_0_) = (0, 0), where θ and φ are the polar and azimuthal angles, respectively. In the model, the pump photon can either be scattered through the sample or be absorbed and give rise to a secondary photon. At each scattering event, the direction of propagation of the photons changed through θ and φ. The absorption probability was set in accordance with the absorption cross section of the sample at 808 nm. The secondary photon was allowed to randomly scatter through the sample with the same initial mean-free-path. The simulation was finished if either the secondary photon was able to reach the surface or if the secondary photon was scattered inside the sample for more than 10,000 times. The simulation was repeated for 5000 pump photons. The ratio between secondary photons that could reach the surface and would be able to be detected (N_e_) to those generated within the sample (N_0_) is shown in Fig. 7, as a function of the transport mean-free-path. The simulation shows that the N_e_/N_0_ ratio is independent of the mean-free-path. Thus, spontaneous emission would not benefit from the reduction of photons mean transport length, that was observed from CBS characterization. For this reason, PL enhancement may be due to the reduction of nonradiative losses, that may be attributed to the incorporation of Nd^3+^ ions into the structure of crystallites that were grown during the annealing treatments. This is corroborated by XRD results, since the Nd_2_Ge_2_O_7_ phase is present in the glass–ceramics even for samples annealed for only 0.5 h. In addition, samples annealed above 3 h, clearly show small sharp features in the PL spectra, which are overlapped to the broad emission band characteristic of the glassy phase. The emerging sharp bands are also an indication that Nd^3+^ ions were incorporated into NdO_7_ and NdO_8_ polyhedra^47^ within the structure of the Nd_2_Ge_2_O_7_ crystallites that were grown during the annealing treatment. The rise on the lifetime after the annealing treatment is also consistent to the fact the Nd^3+^ ions are migrating to specific sites within the crystalline structure of Nd_2_Ge_2_O_7._ In this case, for the parent glass, the average distance among Nd^3+^ ions is probably, much shorter than in the GCs. Thus, the higher proximity of Nd^3+^ ions in the glass enhances the ion-ion interactions causing a reduction in the observed lifetime^48–50^.

### Random laser characterization

We observed and characterized in the present work the photonic analogue behavior of the paramagnetic to spin-glass phase in magnetic materials. The photonic-phase-transition, which corresponds to the transition from the spontaneous emission regime (below the laser threshold) to the RL glassy behavior (above the threshold) was characterized as described in “Optical characterization”.

To analyze the RL modes correlations the Parisi *overlap parameter* ${q}_{\alpha \beta }$ of Eq. (3), discussed in “Optical characterization”, was calculated from *N* = 200 acquired PL spectra, obtained for each energy fluence excitation (EFE) used. The probability distribution *P(q),* $q= {q}_{\alpha \beta }$, was determined for all samples and for various EFEs.

**Figure 8:**
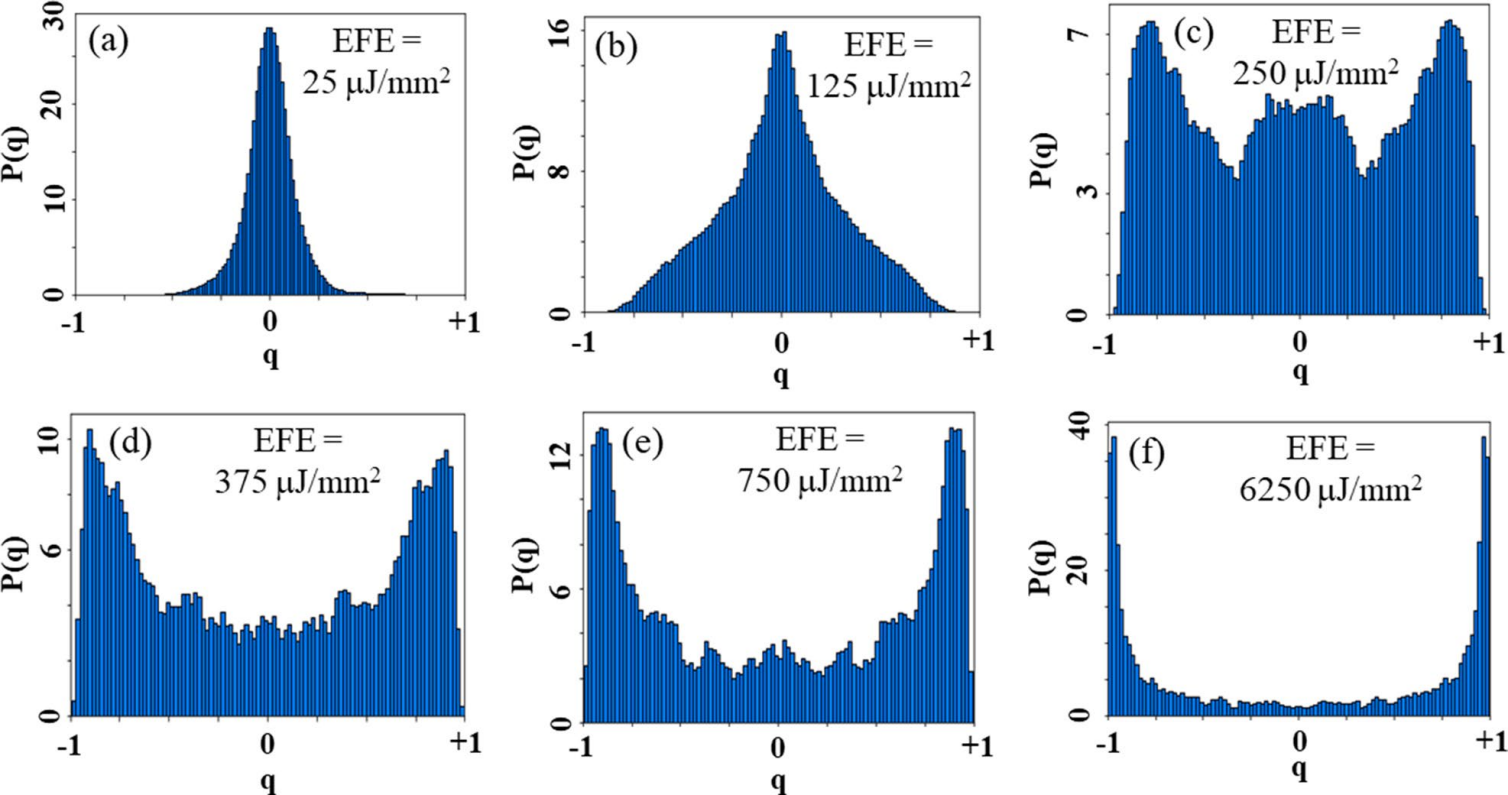
Probability distribution of the Parisi overlap parameter for different EFEs. Below threshold: (**a**) EFE = 25 μJ/mm^2^, and (**b**) EFE = 125 μJ/mm^2^. In both cases the maximum of *P(q)* occurs at q_max_ ≈ 0. (**c**) On the threshold: EFE = 250 μJ/mm^2^; the maximum of *P(q)* occurs at $\left|{\mathrm{q}}_{\mathrm{max}}\right|\cong 1$. Above the RL threshold (**d**) EFE = 375 μJ/mm^2^, (**e**) EFE = 750 μJ/mm^2^ and (**f**) EFE = 6250 μJ/mm^2^; the maximum of *P(q)* consolidates at $\left|{\mathrm{q}}_{\mathrm{max}}\right|\cong 1$.

Figure 8 shows *P(q)* curves obtained for the GPM5 sample. As can be seen in Fig. 8a ${q}_{\mathrm{max}}\approx 0$ when EFE is below the threshold for RL emission (EFE_th_). This indicates absence of correlations among the output intensity fluctuations. As the EFE increases, the shape of *P(q)* changes, as shown in Fig. 8b–f. When EFE $\cong$ EFE_th_, ${q}_{\mathrm{max}}$ shifts to near − 1 and + 1, which indicates correlated and anti-correlated fluctuations, respectively. Nevertheless, a significant number of replicas are not correlated as indicated by the large magnitude of *P(q* = *0)* in Fig. 8c. Above the RL threshold the RSB occurs, and the maximum of *P(q)* consolidates at $\left|{\mathrm{q}}_{\mathrm{max}}\right|\cong 1$, as shown in Fig. 8d–f. It is worth mentioning that the RSB phenomenon was largely reported for different RLs systems^2,37–43^. Yet, it is the first time that RSB phenomenon is reported for a RL based on a glass–ceramic system, for the best of the authors knowledge.

**Figure 9:**
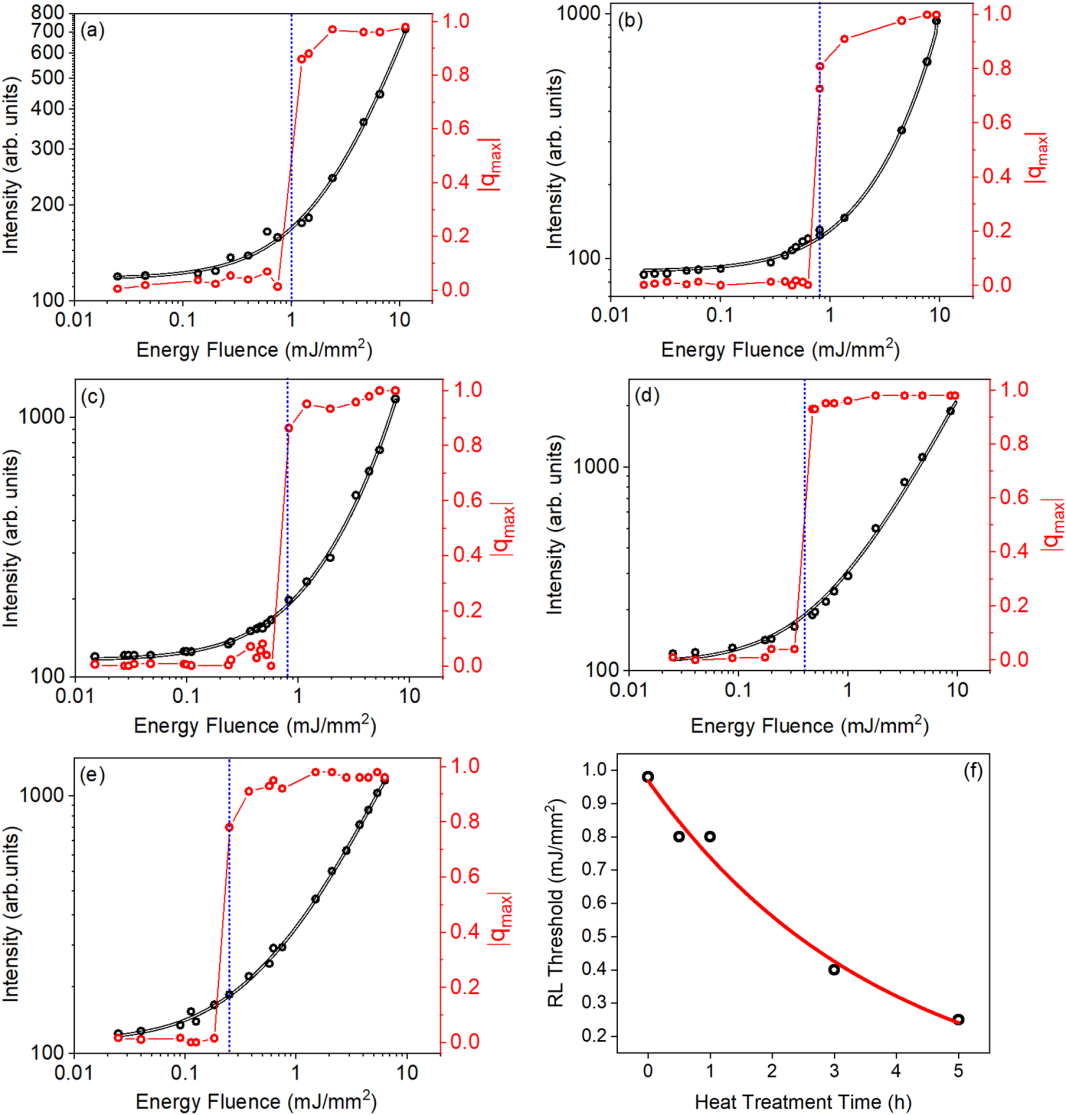
Modulus of the maximum Parisi overlap parameter (red curves) and PL intensity versus EFE at 1068 nm (black curves) for Nd^3+^ doped GPM samples. (**a**) GPM; (**b**) GPM05; (**c**) GPM1; (**d**) GPM3; (**e**) GPM5; (**f**) EFE_th_ as a function of heat treatment time. The dashed blue line indicates the EFE_th_ for each sample.

Figure 9 shows the PL intensity at 1068 nm (black curves) and |${q}_{\mathrm{max}}|$ (red curves) versus the EFE for all samples. A clear transition from spontaneous emission to RL is observed from the PL intensity and |${q}_{\mathrm{max}}|$ curves. Notice that the EFE_th_ obtained from the PL vs EFE curves agrees with the EFE_th_ found for |${q}_{\mathrm{max}}|$ transition. This was also observed for other RLs already reported^2,37–43^.

Observe that EFE_th_ is reduced from ~ 1 to ~ 0.25 mJ/mm^2^ when we compare the untreated glassy GPM sample with the one submitted to the crystallization treatment for 5 h. The results of EFE_th_ as a function of annealing time in Fig. 9f, show clearly that the degree of crystallization is also correlated to the RL performance.

**Figure 10:**
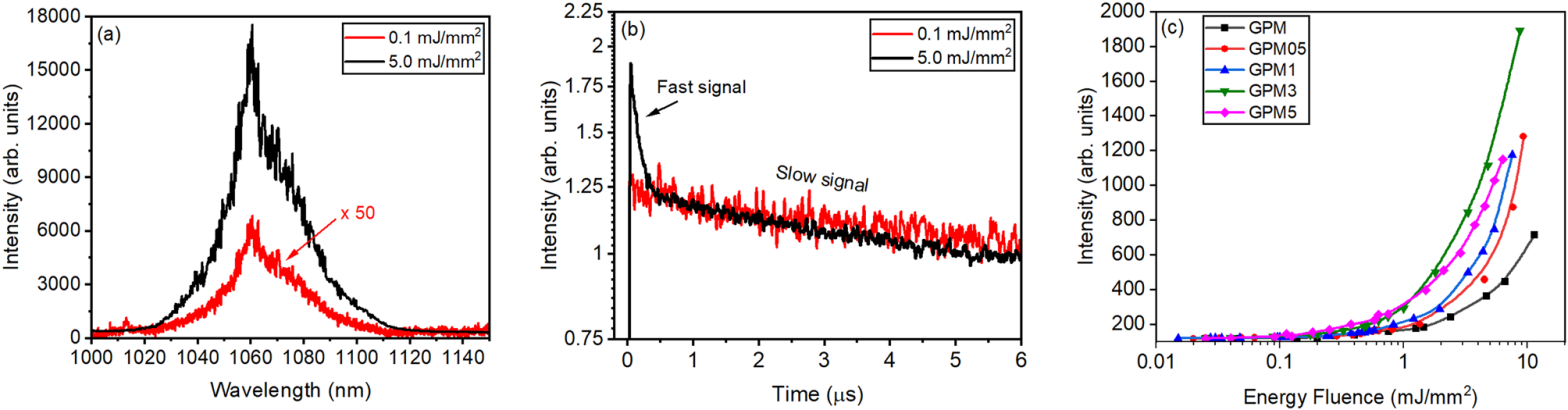
(**a**) Emission spectrum and (**b**) temporal behavior of the signal (at ~ 1068 nm) for the GPM5 sample for EFE below and above EFE_th_. (**c**) PL intensity vs EFE for all samples.

Figure 10a shows that the emission intensity at 1068 nm is about 130 times higher when the EFE is increased from 0.1 to 5.0 mJ/mm^2^, for the GPM5 sample. We did not observe, however, any significant reduction in the width of the emission band. The inhomogeneous broadening is expected for glassy active media and is present in the glass–ceramics because of the residual parent-glass that is still present in the samples, even after heat-treatments. Figure 10b shows the PL dynamics below and above the EFE_th_ for the GPM5 sample. Below EFE_th_, the lifetime was in the μs range for all samples. On the other hand, for EFE > EFE_th_, a fast emission was observed, in the nanosecond range following the pump laser pulse, superimposed on the slower signal (in the µs range) due to the spontaneous emission by the ions that are not participating in the stimulated emission process. The temporal behavior shown by the other samples is like the one shown in Fig. 10b.

In the GPM sample, the RL feedback mechanism is attributed to the micrometric grains. In this case, the glass particles in the powder act simultaneously as scatterers and gain media. Nevertheless, the photon transport length is roughly of the same size as the particles. On the other hand, in the glass–ceramics, crystallites that are grown within the powder grains due to the annealing treatment, may also act as scatterers. Thus, whilst in glass, the photons residence time is governed by light reflections in the particles-air interfaces, in the glass–ceramics, light reflections by the crystallites-glass interfaces may also be relevant. This additional scattering mechanism may increase the photons residence time within the samples reducing the energy fluence threshold for RL observation. In addition, Nd^3+^ inside the crystallites grown within the glass phase may present sharper emission lines and may not suffer from large inhomogeneous broadening^51^. Indeed, Fig. 10c shows that the RL slope-efficiency increases for the samples with a higher crystallinity degree, even though it was not observed reduction on the emission linewidth above EFE_th_. Therefore, despite the inhomogeneous broadening caused by the residual glass, the crystallites act both as scatterers and as more efficient light emitters.

## Summary and conclusions

Nd^3+^ doped GPM glass and glass–ceramics were successfully fabricated, and the crystallization degree of the samples was controlled by a careful annealing treatment at 830 °C for different time intervals. X-ray diffraction analysis showed that the crystallization degree of the samples varied from 0 to 78%, depending upon the heat treatment duration. Nd_2_Ge_2_O_7_ and MgPb_3_Ge_5_O_14_ crystallites were grown in the glass–ceramics because of the annealing treatment. CBS measurements showed that crystallization had, indeed, an impact in the photons mean-free-path that changed from ~ 3.5 µm for the glassy sample to ~ 2.2 µm for the sample heat-treated for 5 h.

Powders consisting of glass and glass–ceramic microparticles were prepared for optical characterization. Approximately 500% of PL enhancement at 1068 nm was observed for samples with a higher crystallinity degree, relative to the glassy sample. We attribute the PL enhancement to the incorporation of Nd^3+^ ions into the structure of the crystallites grown due to the heat treatment of the powders.

The energy fluence excitation threshold (EFE_th_) for RL action was smaller for samples with a higher degree of crystallization. For all samples, we observed replica symmetry breaking (RSB) with the Parisi *overlap parameter* changing from 0 to $\pm 1$ for excitation above the EFE_th_. The RL efficiency was enhanced for the samples that were submitted to higher annealing times. The enhanced RL performance for the glass–ceramics in comparison with the glass samples may be attributed to the residence-time growth of photons inside the powder and by the higher quantum efficiency of Nd^3+^ located in the crystallites because the radiative losses are expected to be smaller than for the ions in the host glass. The optical feedback mechanism for the RL action in the glass–ceramics is attributed to light reflections in the microparticles-air interfaces and the reflections inside the particles with the emitted photons being reflected by the boundaries of the crystallites.

This is the first time, for the best of our knowledge, that the RSB phenomenon is reported for a RL based on rare-earth ions hosted in a glass–ceramics. We claim that investigations of glass–ceramics for random lasers may be a fruitful research field due to the possibility of fine control of the optical feedback by changing the crystallization degree of the samples, which implies that the photons mean-free-path (measured by Coherent Back Scattering) and the RL action in glass–ceramics may be used as tools to investigate the kinetics of crystallization in such materials.

## Methods

### Glass and glass–ceramics fabrication and morphological characterization

The composition of the glasses prepared was 40GeO_2_–55PbO-5MgO (wt%)—labeled as GPM; 10 wt% of Nd_2_O_3_ (corresponding to 1.87 × 10^21^ ions/cm^3^) were added to the final composition. All raw materials used were high purity (> 99.99%). Although it is expected that a large concentration of Nd_2_O_3_ in the glass samples causes luminescence concentration quenching (LCQ), we have already observed that the RL performance is enhanced for higher rare-earth ions concentrations, despite the occurrence of LCQ^30^. The reason is that the dynamic of the RL emission occurs in the nanosecond regime while the PL occurs is in the microsecond range^25,30^. The glasses were obtained by conventional melt-quenching technique. Reagents were melted at 1200 °C in a platinum crucible for 1 h, and then, quenched in water, at room temperature to prevent crystallization. The resulting GPM glasses were ground using a mortar and pestle to obtain a fine powder. Approximately 18 mg of the GPM powder were submitted to Differential Scanning Calorimetry (DSC) analysis (Labsys Evo, Setaram), to verify the most suitable temperatures for the crystallization process. DSC analysis was conducted in N_2_ atmosphere (100 mL/min) using an alumina crucible and the heating rate was 20 °C/min.

The GPM powder was separated in five samples labelled GPM, GPM05, GPM1, GPM3, and GPM5. Each sample was submitted to annealing treatment at 830 °C, in air, for different periods of time, as shown in Table 1. The annealing temperature was slightly below the onset of crystallization that were determined from DSC measurements. After the annealing process, each GC sample was subjected to a second pulverization process. Then, the obtained powders were submitted to X-Ray diffraction (XRD) in a Rigaku SmartLab advanced X-ray diffractometer with Cu K_α_ radiation (λ_x_ = 0.154059 nm; 40 kV; 30 mA) to follow the structural changes of the specimens (step size: 0.01°; time per step: 0.3 s). The average size $(D)$ of crystallites was estimated from the width of X-ray peaks according to Scherrer's equation $D=\frac{K{\lambda }_{x}}{\beta \mathrm{cos}\theta }$^36^, where ${\lambda }_{x}$ is the wavelength of X-ray radiation, $\theta$ is the diffraction angle, $\beta$ is the width of peak at half of its maximum intensity (FWHM), and $K$ is a dimensionless shape factor known as the Scherrer constant (in the case of spherical particles $K$ = 0.94). The percent crystallinity degree $(CD)$ was estimated by the ratio of the crystalline area, ${A}_{C}$, present in the diffractogram of the devitrified glass (glass–ceramics) and the total area, ${A}_{T} (\mathrm{amorphous}+\mathrm{crystalline})$, using the equation $CD =100\frac{{A}_{C}}{{A}_{T}}$^52^.

**Table 1:** Annealing times used in this work for the controlled crystallization of GPM sample (with 10 wt% of Nd_2_O_3_).

Sample	Annealing time (h)
GPM	0.0
GPM05	0.5
GPM1	1.0
GPM3	3.0
GPM5	5.0

In all cases, the annealing temperature was 830 °C.

### Optical characterization

The samples preparation for the optical experiments was according to the following procedure. Initially, each annealed powder was vigorously mixed with isopropyl alcohol to obtain a homogenous suspension. Then, the suspension was left to rest for 30 s for sedimentation of coarser particles. The supernatant was collected, and after solvent evaporation, a fine powder was obtained.

Sample holders were prepared from silica glass microscope slides. Cavities of 10 × 10 mm^2^ and about 500 µm of depth were mechanically etched onto the surface of the glass slides, using silica microspheres sandblasting. The samples’ holders were then cleaned with isopropyl alcohol in an ultrasonic bath. Approximately 100 mg of each powder were placed in the cavity of the sample holders and gently pressed, to obtain homogeneous layers of Nd^3+^ doped GPM glass and GC particles. Other microscope slides were used to cover the cavities containing the samples to avoid powder leakage. Particles size distribution were obtained from optical microscopy images of each sample; the ImageJ open-source software^53^ was used to analyze the micrographs.

The mean-free-path of photons, for each sample, was determined by Coherent Backscattering Scattering (CBS) measurements. The setup for these experiments is described elsewhere^48^. The mean transport path of the photons, ${l}_{t}$, could be obtained from the equation ${l}_{t}=0.7\frac{\lambda }{2\pi W}$^54^, where $\uplambda$ is the laser wavelength (1064 nm) and *W* is the full width at half maximum (FWHM) of the CBS cones.

Diffuse reflectance spectra of the powders were obtained, in the VIS–NIR range, using a spectrophotometer (Ocean Optics), at room temperature. Photoluminescence (PL) experiments were performed at room temperature using two optical sources. For the PL experiments under low pump intensity a CW diode laser operating at 808 nm was used. For experiments with large intensities, it was used an Optical Parametric Oscillator (OPO), OPOTEK INC., Opollete™ HE 532 LD model, pumped by the second harmonic of a Q-switched Nd: YAG laser (7 ns, 20 Hz). The OPO wavelength was tuned to 808 nm, in resonance with the Nd^3+^ transition ^4^I_9/2_ → {^4^F_5/2_, ^2^H_9/2_}, to optimize the fluorescence signal due to the ^4^F_5/2_ → ^4^I_11/2_ transition, leading to a RL emission peak at 1068 nm.

**Figure 11:**
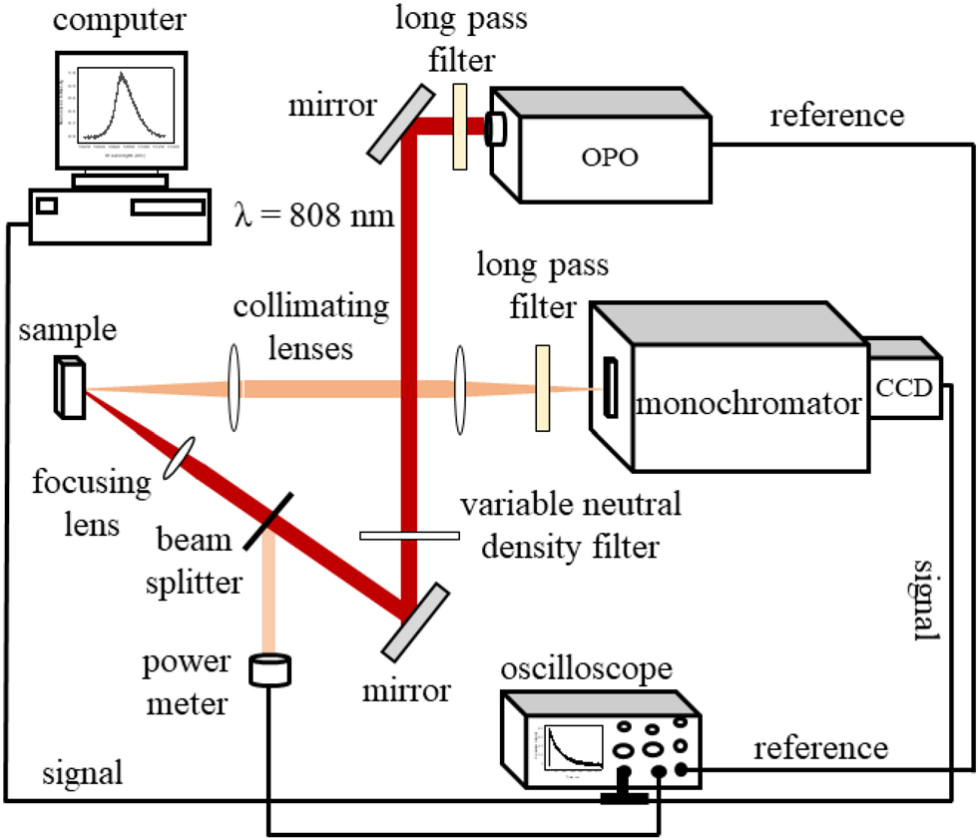
Setup for random laser characterization.

The light beam from the OPO was focused on the sample by a 70 mm focal length lens, corresponding to an illuminated area of about 200 µm of diameter. The angle between the perpendicular direction to the sample face and the incident laser beam was 45° and the scattered light emitted from the sample was collected from its front surface and focused into a high-resolution (~ 0.01 nm) spectrometer coupled to a charge-coupled device (CCD). A long-pass filter was positioned at the entrance of the monochromator to remove the scattered light due to the incident laser beam. The output intensity was controlled by a variable neutral density filter, and a reference of the input intensity was monitored by a photodiode coupled to an oscilloscope. PL intensity versus the Energy Fluence Excitation (EFE) curves were obtained by tuning the center of the monochromator grating to the maximum emission intensity ($\approx$ 1068 nm), and then, 200 spectra were obtained for each pumping condition to plot PL intensity vs EFE curves. The critical energy fluence for the transition between spontaneous emission to RL regime (EFE_th_) could be determined from the PL intensity versus EFE curves. The PL temporal evolution was determined using the same setup as described above but changing the CCD by an IR photomultiplier coupled to a 2 GHz bandwidth model oscilloscope (Tektronix MSO5204B Mixed Signal). Figure 11 illustrates the experimental setup used to characterize the RL emission.

The *photonic phase-transition* and the associated phenomenon of RSB were observed and characterized using the PL spectra. The probability density function, *P(*${q}_{\alpha \beta }$*)*, where ${q}_{\alpha \beta }$ is called the *overlap parameter*, measures the degree of correlation among the RL spectral modes being determined for EFEs below and above the RL threshold^2,37,40–43^. The values of ${q}_{\alpha \beta }$ were obtained considering the intensity fluctuations by using the expression^37,40–43^.

3 $${q}_{\alpha \beta }= \frac{\sum_{k}{\Delta }_{\alpha }(k){\Delta }_{\beta }(k)}{\sqrt{\left[\sum_{k}{\Delta }_{\alpha }^{2}(k)\right]\left[\sum_{k}{\Delta }_{\beta }^{2}(k)\right]}} ,$$

where $\alpha$*,* $\beta$ = *1*, *2*,…, *N* denote the replica labels; the average intensity at the wavelength indexed by *k* is represented by $\langle I\rangle \left(k\right)=\sum_{\alpha =1}^{N}{I}_{k}(k)/N$, and the intensity fluctuation is symbolized by ${\Delta }_{\alpha }\left(k\right)={I}_{\alpha }\left(k\right)-\langle I\rangle \left(k\right)$. Each output spectrum is considered a replica, i.e., a copy of the RL system under initial identical experimental conditions. Then, the distribution *P(*${q}_{\alpha \beta }$*)* can be determined for each value of the EFE as in references^37–43^.

## Data Availability

The datasets used and/or analysed during the current study are available from the corresponding author on request.
